# Realistic simulation of syphilis investigation during pregnancy in public health nursing education

**DOI:** 10.1590/0034-7167-2024-0392

**Published:** 2025-11-28

**Authors:** Juliana Moura Lamblet Severo Costa, Anna Carolina Hiromi Royer Uemura, Marcos Morais Santos Silva, Mayara Maria Souza de Almeida, Érica Gomes Pereira, Lúcia Yasuko Izumi Nichiata

**Affiliations:** I Universidade de São Paulo. São Paulo, São Paulo, Brazil

**Keywords:** Educational Measurement, Syphilis, Nursing Research, Simulation Training, Public Health., Evaluación Educacional, Sífilis, Investigación en Enfermería, Entrenamiento Simulado, Salud Pública.

## Abstract

**Objectives:**

to assess a realistic simulation lesson plan on syphilis investigation during pregnancy for nursing courses from undergraduate students’ perspective.

**Methods:**

this is a methodological research that assesses a lesson plan based on cognitive, affective and psychomotor domains, comprising the following stages: content identification; prebriefing; briefing; scenario; assessment by experts; application of the plan with students in the 7^th^ semester of the undergraduate nursing course at a public university located in the city of São Paulo; and application of a questionnaire to assess the content, self-confidence and perception in the learning process.

**Results:**

the plan was validated by students, evidencing significant educational gain, highlighting the importance of prebriefing and post-simulation discussion for learning.

**Conclusions:**

the lesson plan was considered effective and contributed to learning in the investigation of syphilis in pregnancy.

## RESUMEN

**Objetivos:**

evaluar un plan de lección de simulación realista sobre la investigación de la sífilis durante el embarazo para cursos de enfermería desde la perspectiva de estudiantes de pregrado.

**Métodos:**

se trata de una investigación metodológica que evalúa un plan de lección basado en las áreas cognitiva, afectiva y psicomotora. Este plan comprende las siguientes etapas: identificación del contenido; *prebriefing; briefing*; escenario; evaluación por expertos; aplicación del plan con estudiantes de séptimo semestre de la carrera de enfermería de una universidad pública de São Paulo; y aplicación de un cuestionario para evaluar el contenido, la autoconfianza y la percepción del proceso de aprendizaje.

**Resultados:**

el plan fue validado por los estudiantes, lo que evidenció un avance educativo significativo, destacando la importancia de la *prebriefing* y la discusión posterior a la simulación para el aprendizaje.

**Conclusiones:**

el plan de clase se consideró eficaz y contribuyó al aprendizaje en la investigación de la sífilis en el embarazo.

## INTRODUCTION

Untreated syphilis in pregnant women is a major health problem because it can lead to congenital syphilis (CS). CS can cause damage to fetal development, including the involvement of various organs and systems, which can lead to miscarriage, stillbirth, and infant death^(1)^; therefore, its elimination remains a global challenge.

The World Health Organization (WHO) targets for 2030 indicate reducing the number of CS cases to less than 50 cases per 100,000 live births in 80% of the world’s countries^(2)^. According to the Pan American Health Organization (PAHO), the proposed recommendation for eliminating vertical transmission of HIV and syphilis in the Latin American region consists not only of increasing prenatal care and deliveries in hospital settings by more than 95%, but also of increasing testing of pregnant women and treatment of those who are seropositive for syphilis by more than 95%^(3)^.

In Brazil, in 2023, the syphilis detection rate in pregnant women was 34.0 cases per 1,000 live births, indicating a significant increase in recent years (2013-2023)^(4)^. The Southeast, Central-West and South regions presented rates higher than the national average, with emphasis on the state of Rio de Janeiro, which recorded the highest rate of syphilis detection in pregnant women, with 69.5 cases per 1,000 live births^(1)^, showing the importance of well-performed prenatal care.

Early diagnosis of syphilis during pregnancy, through adequate prenatal care, and treatment of pregnant women with benzathine benzylpenicillin^(1)^ at the correct dose for each stage of syphilis’s progression are proven effective in preventing vertical transmission of T. *pallidum*, and have been used as a recommendation for CS prevention since 1950^(2)^. It has a broad cost-effectiveness ratio, and is an effective way to prevent and control syphilis, reducing costs arising from complications of syphilis^(1)^. A fundamental part of screening is carried out through investigation of suspected syphilis in pregnancy by nursing, particularly carried out in Primary Care^(2)^.

Therefore, there are effective measures for syphilis prevention and treatment that can be offered to pregnant women and newborns to prevent the development of syphilis. However, there are challenges to be faced in the investigation of syphilis during pregnancy, such as improving the quality and care taken in filling out the notification form, as well as acquiring knowledge and correctly using case assessment criteria during screening in nursing consultations.

In order to achieve the PAHO and WHO objectives, the investigation of syphilis during pregnancy should be included as a skill to be developed in undergraduate nursing education, in which the use of active methodologies has been an alternative to support learning^(5)^. Realistic simulation is a pedagogical strategy that provides undergraduate students with the tools to understand how an investigation of syphilis during pregnancy can occur in practice in the present project. Among the advantages of teaching based on realistic simulation, it is possible to mention the fusion of theoretical and practical content and the development of clinical reasoning for the investigation of complex and individualized cases^(6)^. Realistic simulation can be developed in laboratory environments and simulation centers, to ensure a scenario for reflection combined with the development of care skills for care centered on health users^(7)^.

## OBJECTIVES

To assess a realistic simulation lesson plan on syphilis investigation during pregnancy for nursing courses from undergraduate students’ perspective.

## METHODS

### Ethical aspects

The study was conducted in accordance with national and international ethics guidelines and was approved by the *Universidade de São Paulo* School of Nursing (EEUSP) Research Ethics Committee, respecting the ethical precepts set forth in the standards of Resolution 466/12 of the National Health Council. Acceptance to participate in the study occurred through signing the Informed Consent Form, which was made available and obtained from all individuals involved in the study in writing. Furthermore, the study was guided by the General Data Protection Law 13,709/2018.

### Study design, period and place

This is methodological research to assess a realistic simulation lesson plan, based on the International Nursing Association for Clinical Simulation and Learning recommendations^(8)^. It was carried out in five phases (overview, scenario, scenario design progression, debriefing, and assessment), in order to guarantee accuracy and transparency in the simulation scenario validity on the investigation of syphilis in pregnancy. The study was carried out in the city of São Paulo, at EEUSP, and was developed from March 2023 to March 2024.

### Study population: inclusion and exclusion criteria

The study population consisted of five judges, including professors and specialists in syphilis and public health, who were responsible for assessing the realistic simulation lesson plan. Judges with at least two years of experience in investigating syphilis during pregnancy, working in different educational and health institutions, and didactic experience in teaching syphilis or epidemiological surveillance of CS, with specialization in realistic simulation, were included. Judges were selected based on the *Lattes* CV of professors at EEUSP and *Universidade Estadual Paulista - Botucatu*, who have expertise in the subject.

In addition, 33 students, who assessed and attended the simulation scenario, participated in the study. Students aged 18 or older and enrolled in the “Nursing in Communicable Diseases with a Focus on Public Health” discipline, offered in the 7^th^ semester of the undergraduate course at EEUSP, were included. Students who did not participate in the theoretical class on syphilis or were away from academic activities during data collection were excluded from the sample.

### Study protocol

The lesson plan was based on Bloom’s theory, which helps professors organize and improve the educational process. The theory covers three domains - cognitive, affective and psychomotor - which were considered in the plan development^(9)^.

The cognitive domain addresses students’ ability to assign practical meaning to information, divided into six hierarchical levels: knowledge; understanding; application; analysis; synthesis; and assessment. The affective domain involves emotional and behavioral aspects of learning, organized into five levels: reception; response; assessment; organization; and characterization. The psychomotor domain uses physical skills to acquire knowledge, divided into perception, predisposition, guided response, mechanical response, and complete response^(6)^. In the context of syphilis investigation during pregnancy, it is essential that students recognize, analyze, apply and assess previous information before approaching new content.

### Realistic simulation lesson plan stages after expert assessment

1. Overview: theoretical presentation on the CS health-disease process, addressing the recognition and investigation of syphilis in pregnant women, considering the stage of syphilis, technical skills of prevention and treatment, and social aspects related to epidemiology. This stage was carried out in a theoretical class. Prebriefing: held the day before the session, with guidance on the simulated environment and resources for scene progression. The lesson plan includes previously prepared support materials, such as a scenario script, fictitious medical records, prenatal card and situational progression script for the actor (actress).
2. Scenario (moment before the scenario): the objective was to recall processes, define objectives and collect information for syphilis investigation simulation during pregnancy, occurring in three parts. Part 1: welcome, with reception of participants, presentation of the objective of the class and selection of two volunteers (simulated nurse and resident). Part 2: scenario environment assessment by all. Part 3: guidance on the roles of each participant: a) simulated nurse: medical record consultation (notes, genogram and ecomap), case problem, government documents and preparation of care; b) nurse in training: study of medical record, case problem and checklist analysis; c) simulated user: medical record and history consultation, following the action flowchart.

### Case problem: for reading by participants and volunteers

Karine, 25 years old, white, heterosexual, married, mother of two children, 29 weeks pregnant, with incomplete elementary school and unemployed. She went to the Basic Health Unit for her third prenatal appointment. During physical examination, Karine told the nurse that she noticed the reappearance of the wound on her anus, which she had reported at her previous prenatal appointment (three months ago). She reported that she had a lesion in the perianal region and some “red spots” on her hands, and among the laboratory tests performed, a syphilis test was not requested. The only syphilis test was performed at 14 weeks, which resulted in “non-reactive”. In addition, she mentioned that her husband has a history of extramarital affairs. When asked about the type of sexual practice, she stated that she only has vaginal, anal and oral sex with her husband, without using a condom. You and the resident nurse will provide care to the user.

3. Scenario design progression: scenario progression - represented not only the environment to be simulated, but the entire theoretical-practical construction to be planned, executed and assessed with the greatest degree of detail and realism possible with the available resources. For the scenario, the volunteer or randomly selected students carry out a consultation to investigate syphilis during pregnancy with the researchers acting in the scenarios as actresses. The other students, when observing the scene, made observations and notes about the care provided, considering the previous learnings in the class on syphilis in pregnancy, with the help of a checklist designed to identify the main characteristics and investigation actions expected in consultation. Scenario progression is led by the simulated user with a flow of responses and actions to be performed, previously agreed with the flowchart located in Figure 1. During the moment after simulation, students brought their perceptions about the case, doubts, feedback and what they would do differently, generating a joint construction of learning. Then, observers comment on the checklist:
  **Figure 1:**
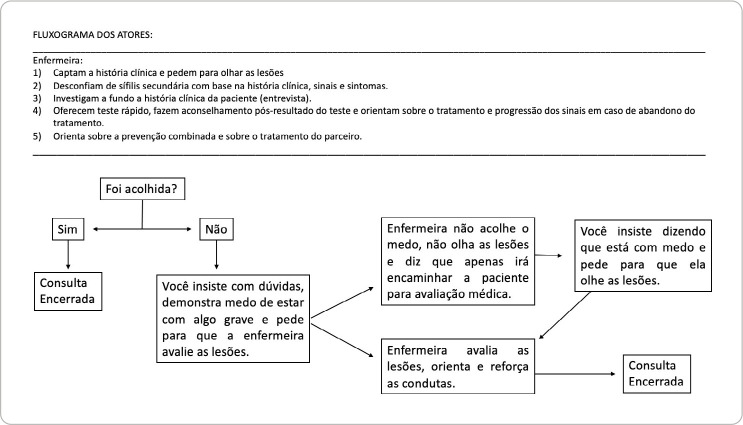
Scenario progression flowchart: the simulated user uses the flow of actions and responses to define the scenario progression and end

**Checklist -** Verification for simulated nurses in training with the following items: posture: assesses cordiality and considers users’ safety protocol; communication: assesses the use of simple and clear language, providing guidance on consultation progression and promoting a space for active listening, clear guidance and support for the problems and concerns presented; interview: asks about living, work, housing and health conditions? Do they ask about sexual practices, daily and cultural habits? Do they ask about partnerships, prevention of sexually transmitted infections and use of contraceptives? Do they respect users’ cultural aspects?; physical examination: do they inspect and assess the skin, mucous membranes, lesions, and perform a physical examination aimed at prenatal care? Do they ask and provide guidance on hygiene procedures? Do they perform testing? and conduct: do they provide guidance on nursing consultations to monitor lesions, on treatment and self-care? Do they offer rapid tests? Do they recommend the VDRL test to users and contacts? Do they praise and encourage progress in self-care? Do they establish self-care goals? Do they provide guidance on treatment and prevention? Do they prescribe penicillin treatment? Do they fill out the syphilis notification form?

4. Debriefing: assessment - the lesson plan was tested with a group of experts composed of professors, specialists, nurses and students, and was previously assessed by the judge committee and then applied to be assessed by students. After the discussion, together with volunteer students and observers, participants assessed the simulation and the lesson plan using the semi-structured questionnaire.
5. Assessment: the simulation lesson plan was carried out with 7^th^ semester students, accompanied by the application of a semi-structured questionnaire (Likert scale) to assess content, self-confidence and perception in the research process. The activity, including presentation, simulation and filling out of the questionnaires, lasted approximately 120 minutes.

Questionnaires addressed to the expert committee were used as data collection instruments, divided into two parts: 1) sociodemographic data; and 2) items on simulation and content technical aspects. With an average duration of 30 minutes, the questionnaires were completed during the pilot test, before being administered to students. Inspired by a study on the validity of teaching materials for syphilis prevention and management^(5)^, the questionnaires assessed the teaching method using the Likert scale, with response options ranging from “strongly disagree” to “strongly agree”.

A sociodemographic questionnaire prepared by undergraduate students was administered. The teaching method was assessed using questionnaires based on the Likert scale, which were handed out at the end of simulation^(10)^. The student questionnaire addressed the simulation structure, content, and participants’ self-confidence.

## RESULTS

The expert committee positively assessed all topics of the simulated class on syphilis investigation during pregnancy. Everyone agreed that the simulation addresses content relevant to the topic and that the dynamic format can significantly contribute to clinical and epidemiological learning. The previous content was considered relevant, and the simulated scenario was assessed as pleasant, informative, coherent and appropriate. They also mentioned that prebriefing and debriefing are well detailed, being crucial stages for understanding and learning the topic. Such detail, observed in all the material validated by the expert committee, helps to break taboos and does not foster embarrassment related to the topic in nursing consultation.

When assessing students’ satisfaction and self-confidence after realistic simulation, ten questions were asked, and the result was: 63.6% (21) of participants fully agreed that they felt confident in dealing with the investigation of syphilis during pregnancy; 87.9% (29) fully agreed that the activity contributed significantly to their own training and the understanding of content about the investigation of syphilis during pregnancy; 78.7% (26) found the simulation enjoyable; 69.7% (23) disagreed that they preferred traditional learning activities; 90.9% (30) agreed that they were able to develop skills through simulation activities; 90.9% (30) fully agreed that initial explanation, before the scenario, was important for understanding the dynamics; 84.8% (28) fully agreed that the simulation discussion was efficient in the learning process; 93.9% (31) agreed that the simulation helped break taboos regarding the nursing consultation theme addressed; and 100% (33) agree that their knowledge about syphilis investigation during pregnancy improved after the simulation. The only question that received different answers was about the broad knowledge about syphilis investigation before simulation, in which we obtained 60.6% (20) who disagreed, 12.1% (4) who did not know how to answer and 27.3% (9) who agreed.

The sample profile presents a composition of a white majority, with cisgender female identification, equally divided between heterosexuals and bisexuals, assuming exclusive occupation as a student, according to the sociodemographic survey completed by participants.

## DISCUSSION

The realistic simulation lesson plan was validated by a committee of experts and tested by students, highlighting the importance of practical activities with active learning methodologies in nursing education. The simulation addressed the investigation of syphilis during pregnancy, essential for the development of students’ clinical reasoning, contributing to early detection and appropriate treatment, preventing complications such as CS.

In addition to technical aspects, the simulation provided reflections on social and public health issues, such as inequalities in access to care and information. The study points out that, by focusing only on technical treatment, nursing can neglect the social implications of syphilis, while effective clinical investigation, with good anamnesis and risk assessment, is essential to prevent problems for pregnant women and babies.

During debriefing, students discussed nursing autonomy, especially in relation to ordering tests and prescribing medications. The discussions indicated that the consolidation of autonomy and clinical decision-making skills is still under development. Simulation helped participants reflect on their practices and limitations in Primary Health Care, suggesting the need for further studies on student satisfaction and self-confidence in these scenarios.

An innovative aspect of simulation was researchers’ participation, who were also students, acting as simulated users. This provided a more comfortable and collaborative environment, encouraging students to share their opinions and discuss their decisions. Debriefing was crucial for critical reflection and consolidation of learning, with professors’ contribution as facilitators of the scenario, clarifying doubts and enriching the teaching process.

### Study limitations

Study limitations include the fact that it was conducted in a single public higher education institution, with a small sample size and with a secondary focus on completing the mandatory notification form. In this regard, the results found cannot be generalized to nursing students with characteristics different from our population. The instrument for capturing student satisfaction and self-confidence in learning was used only after participation in realistic simulation.

### Contributions to nursing

This study contributed to developing nursing students’ knowledge about syphilis investigation during pregnancy, improving the quality of epidemiological investigation, and training future nurses to perform high-quality nursing consultations in the screening and management of gestational syphilis, using realistic simulation. In the long term, this may contribute to increasing the early detection of syphilis during pregnancy and improving syphilis prevention and treatment, reducing CS cases and thus responding to the WHO objectives.

## CONCLUSIONS

The lesson plan developed and proposed was assessed, allowing students to obtain a better understanding of the topic in a practical way. Hence, its contribution was highlighted and can be used as material for elaborating teaching materials focused on communicable diseases and investigation of syphilis during pregnancy. The proposal to apply an active methodology in nursing education is promising and should be encouraged, with the goal of eliminating CS, in accordance with the Sustainable Development Goals. The realistic simulation applied to the investigation of syphilis during pregnancy is a method of great didactic and academic use.

## Data Availability

The research data are available only upon request.
