# Supplementary material for: Realistic simulation of syphilis investigation during pregnancy in public health nursing education
Source: Rev Bras Enferm. 2025 Nov 28;78(5):e20240392. doi: 10.1590/0034-7167-2024-0392 (PMC12673608; doi:10.1590/0034-7167-2024-0392)
Supplement: Translation

# Translation: Simulação realística de investigação de sífilis na gestação no ensino de enfermagem em saúde coletiva

**DOI:** 10.1590/0034-7167-2024-0392pt

**Authors:** Juliana Moura Lamblet Severo Costa, Anna Carolina Hiromi Royer Uemura, Marcos Morais Santos Silva, Mayara Maria Souza de Almeida, Érica Gomes Pereira, Lúcia Yasuko Izumi Nichiata

**Affiliations:** I Universidade de São Paulo. São Paulo, São Paulo, Brasil

**Keywords:** Avaliação Educacional, Sífilis, Pesquisa em Enfermagem, Treinamento por Simulação, Saúde Coletiva.

## Abstract

**Objetivos:**

avaliar um plano de aula de simulação realística sobre investigação de sífilis na gestação para cursos de enfermagem na perspectiva de estudantes de graduação.

**Métodos:**

trata-se de pesquisa metodológica que avalia um plano de aula com base em domínios cognitivo, afetivo e psicomotor, compreendendo as seguintes etapas: identificação de conteúdo; pré-*briefing; briefing*; cenário; avaliação por especialistas; aplicação do plano com estudantes do 7º semestre do curso de graduação em enfermagem de universidade pública localizada na cidade de São Paulo; e aplicação de questionário de avaliação do conteúdo, autoconfiança e percepção no processo de aprendizagem.

**Resultados:**

o plano foi validado pelos estudantes, evidenciando ganho educativo significativo, destacando importância do pré-*briefing* e da discussão pós-simulação para o aprendizado.

**Conclusões:**

o plano de aula foi considerado eficaz e contribuiu para o aprendizado na investigação de sífilis na gestação.

## INTRODUÇÃO

A sífilis na pessoa grávida não tratada é um importante problema de saúde por sua possibilidade de determinar a sífilis congênita (SC). A SC pode provocar danos no desenvolvimento fetal, como o comprometimento de diversos órgãos e sistemas, que podem acarretar em aborto, natimortalidade e óbito infantil^(1)^; por isso, sua eliminação permanece como um desafio mundial.

As metas da Organização Mundial da Saúde (OMS) para 2030 indicam a redução do número de casos de SC inferior a 50 casos por 100.000 nascidos vivos em 80% dos países do mundo^(2)^. Segundo a Organização Panamericana de Saúde (OPAS), a recomendação proposta para a eliminação da transmissão vertical do HIV e sífilis na região da América Latina consiste não apenas em aumentar o cuidado do pré-natal e dos partos em ambientes hospitalares em mais de 95%, mas também em aumentar a testagem de gestantes e o tratamento das soropositivas para sífilis em mais de 95%^(3)^.

No Brasil, em 2023, a taxa de detecção de sífilis em gestantes foi de 34,0 casos por 1.000 nascidos vivos, indicando um aumento significativo nos últimos anos (2013-2023)^(4)^. As regiões Sudeste, Centro-Oeste e Sul apresentaram taxas superiores à média nacional, com destaque para o estado do Rio de Janeiro, que registrou a maior taxa de detecção de sífilis em gestantes, com 69,5 casos por 1.000 nascidos vivos^(1)^, mostrando a importância de um pré-natal bem realizado.

O diagnóstico precoce da sífilis na gestação, por meio de pré-natal adequado, e o tratamento da pessoa que gesta com benzilpenicilina benzatina^(1)^ na dose correta para cada estágio de evolução da doença são comprovadamente efetivos para impedir a transmissão vertical do T. *pallidum*, e é utilizado como recomendação para a prevenção da SC desde 1950^(2)^. Possui ampla relação de custo-efetividade, e são formas eficazes para a prevenção e controle da doença, reduzindo os gastos provenientes das complicações da sífilis^(1)^. Parte fundamental do rastreamento é realizada por meio da investigação de suspeição da sífilis na gravidez pela enfermagem, particularmente realizada na Atenção Primária^(2)^.

Há, portanto, medidas eficazes de prevenção e tratamento da sífilis a serem ofertadas à gestante e ao recém-nascido para evitar o desenvolvimento da doença. No entanto, há desafios a serem enfrentados no âmbito da investigação da sífilis na gestação, como a melhoria da qualidade e do cuidado no preenchimento da ficha de notificação, bem como a aquisição de conhecimento e o uso correto dos critérios de avaliação de casos durante o rastreio nas consultas de enfermagem.

Com a finalidade de atingir os objetivos propostos pela OPAS e pela OMS, a investigação de sífilis na gestação deve constar como competência a ser desenvolvida no ensino de graduação em enfermagem, no qual o uso de metodologias ativas tem sido uma alternativa de apoio ao aprendizado^(5)^. A simulação realística é uma estratégia pedagógica que instrumentaliza os estudantes de graduação na compreensão de como pode ocorrer na prática, no presente projeto, uma investigação de sífilis na gestação. Entre as vantagens do ensino baseado em simulação realística, é possível citar a fusão dos conteúdos teóricos aos práticos e o desenvolvimento de raciocínio clínico para a investigação de casos complexos e individualizados^(6)^. A simulação realística pode ser elaborada em ambientes de laboratórios e em centros de simulação para garantir um cenário de reflexão aliado ao desenvolvimento de competências assistenciais para um cuidado centrado no usuário de saúde^(7)^.

## OBJETIVOS

Avaliar um plano de aula de simulação realística sobre investigação de sífilis na gestação para cursos de enfermagem na perspectiva dos estudantes de graduação.

## MÉTODOS

### Aspectos éticos

O estudo foi conduzido de acordo com as diretrizes de ética nacionais e internacionais, sendo aprovado pelo Comitê de Ética em Pesquisa da Escola de Enfermagem da Universidade de São Paulo (EEUSP), respeitando os preceitos éticos dispostos nas normas da Resolução n^o^ 466/12 do Conselho Nacional de Saúde. O aceite para participar do estudo ocorreu por meio da assinatura do Termo de Consentimento Livre e Esclarecido, que foi disponibilizado e obtido de todos os indivíduos envolvidos no estudo por meio escrito. Além disso, o estudo tomou por baliza a Lei Geral de Proteção de Dados nº 13.709/2018.

### Desenho, período e local do estudo

Trata-se de pesquisa metodológica para avaliação de um plano de aula de simulação realística, tomando por base as recomendações da *International Nursing Association for Clinical Simulation and Learning* ^(8)^. Foi realizado em cinco fases (*overview, scenario, scenario design progression, debriefing* e *assessment*), de modo a garantir a precisão e transparência na validação do cenário de simulação sobre a investigação de sífilis na gestação. O estudo foi realizado no município de São Paulo, na EEUSP, tendo sido desenvolvido no período de março de 2023 a março de 2024.

### População do estudo: critérios de inclusão e exclusão

A população do estudo foi composta por cinco juízes, sendo docentes e especialistas em sífilis e saúde coletiva, responsáveis por avaliar o plano de aula da simulação realística. Os critérios de inclusão dos juízes foram ter pelo menos dois anos de experiência na investigação de sífilis na gestação, atuar em diferentes instituições de ensino e saúde, e ter experiência didática em ensino de sífilis ou vigilância epidemiológica de SC, com especialização em simulação realística. Os juízes foram selecionados com base no Currículo *Lattes* de docentes da EEUSP e da Universidade Estadual Paulista - Botucatu, que possuem expertise no tema.

Além disso, participaram do estudo 33 estudantes, que avaliaram e participaram do cenário de simulação. Estudantes com 18 anos ou mais e matriculados na disciplina “Enfermagem em Doenças Transmissíveis com Enfoque em Saúde Coletiva”, oferecida no 7º semestre da graduação na EEUSP, foram incluídos. Estudantes que não participaram da aula teórica sobre sífilis ou estavam afastados das atividades acadêmicas durante a coleta de dados foram excluídos da amostra.

### Protocolo do estudo

O plano de aula foi baseado na teoria de Bloom, que auxilia os docentes na organização e melhoria do processo educacional. A teoria abrange três domínios - cognitivo, afetivo e psicomotor -, que foram considerados na elaboração do plano^(9)^.

O domínio cognitivo aborda a capacidade do estudante de atribuir significado prático às informações, dividindo-se em seis níveis hierárquicos: conhecimento; compreensão; aplicação; análise; síntese; e avaliação. O domínio afetivo envolve aspectos emocionais e comportamentais no aprendizado, organizados em cinco níveis: recepção; resposta; avaliação; organização; e caracterização. Já o domínio psicomotor utiliza habilidades físicas para adquirir conhecimento, dividido em percepção, predisposição, resposta guiada, resposta mecânica e resposta completa^(6)^. No contexto da investigação de sífilis na gestação, é essencial que o estudante reconheça, analise, aplique e avalie informações prévias antes de abordar novos conteúdos.

### Etapas do plano de aula de simulação realística após avaliação pelos especialistas

1. *Overview:* apresentação teórica sobre o processo de saúde-doença da SC, abordando o reconhecimento e investigação da sífilis em gestantes, considerando o estágio da doença, as competências técnicas de prevenção e tratamento, e os aspectos sociais relacionados à epidemiologia. Essa etapa foi realizada em uma aula teórica. Pré-*briefing:* realizado no dia anterior à sessão, com orientações sobre o ambiente simulado e recursos de progressão da cena. O plano de aula inclui materiais de apoio previamente elaborados, como roteiro do cenário, prontuário fictício, cartão de pré-natal e roteiro de progressão situacional para o(a) ator(atriz).
2. *Scenario* (momento anterior ao cenário): teve como objetivo relembrar processos, definir objetivos e coletar informações para a simulação da investigação de sífilis na gestação, ocorrendo em três partes. Parte 1: boas-vindas, com recepção dos participantes, apresentação do objetivo da aula e escolha de dois voluntários (enfermeiro(a) simulado(a) e residente). Parte 2: avaliação do ambiente do cenário por todos. Parte 3: orientação sobre os papéis de cada participante: a) enfermeiro(a) simulado(a): consulta ao prontuário (anotações, genograma e ecomapa), caso-problema, documentos governamentais e preparação do atendimento; b) enfermeiro(a) em treinamento: estudo do prontuário, caso-problema e análise do *checklist*; c) usuária simulada: consulta ao prontuário e histórico, seguindo o fluxograma de atuação.

### Caso-problema: para leitura dos participantes e voluntários

Karine, 25 anos, mulher branca, heterossexual, casada, mãe de dois filhos, gestante de 29 semanas, com ensino fundamental incompleto e desempregada. Compareceu à Unidade Básica de Saúde para realizar a terceira consulta de pré-natal. Durante o exame físico, Karine relatou à enfermeira que observou o reaparecimento da ferida no ânus, relatado em sua consulta anterior de pré-natal (há três meses). Ela informou que possuía uma lesão na região perianal e algumas “manchas vermelhas” nas mãos, e entre os exames laboratoriais realizados, o teste de sífilis não foi solicitado. O único teste de sífilis foi realizado com 14 semanas, cujo resultado foi “não reagente”. Além disso, mencionou que seu marido tem histórico de relacionamentos extraconjugais. Quando questionada sobre o tipo de prática sexual, afirmou que pratica sexo vaginal, anal e oral apenas com seu marido, sem utilizar preservativo. Você e a(o) enfermeira(o) residente irão fazer o atendimento da usuária.

3. *Scenario design progression:* progressão do cenário - representou não só o ambiente a ser simulado, mas toda a construção teórico prática a ser planejada, executada e avaliada com o maior grau de detalhamento e realismo possível com os recursos disponíveis. Para o cenário, os estudantes voluntários ou sorteados realizam uma consulta de investigação para sífilis na gestação com atuação das pesquisadoras nos cenários como atrizes. Os demais estudantes, ao observar a cena, fizeram observações e apontamentos sobre o atendimento considerando os aprendizados anteriores na aula sobre sífilis na gestação com auxílio de *checklist* elaborado para identificar as principais características e ações de investigação esperadas na consulta. A progressão do cenário é conduzida pela usuária simulada com um fluxo de respostas e ações a serem executadas, previamente combinadas com o fluxograma localizado na Figura 1. Durante o momento após a simulação, os estudantes trouxeram suas percepções sobre o caso, dúvidas, *feedbacks* e o que fariam de forma diferente, gerando uma construção conjunta de aprendizado. Em seguida, os observadores comentam sobre o *checklist:*
  **Figura 1:** Fluxograma de progressão do cenário: a usuária simulada utiliza o fluxo de ações e respostas para definir a progressão e fim do cenário

**Checklist -** Verificação para enfermeiros(as) simulados(as) em treinamento com os seguintes itens: postura: avalia cordialidade e contempla o protocolo de segurança da usuária; comunicação: avalia uso de linguagem simples e clara, orientando sobre a progressão da consulta e promovendo espaço de escuta ativa, orientações claras e acolhimento para os problemas e preocupações apresentados; entrevista: questiona sobre condições de vida, trabalho, moradia e saúde? Questiona sobre práticas sexuais, hábitos diários e culturais? Questiona sobre parcerias, prevenção de infecções sexualmente transmissíveis e uso de contraceptivos? Respeita aspectos culturais da usuária?; exame físico: realiza inspeção e avaliação de pele, mucosa, lesões, e exame físico direcionado ao pré-natal? Questiona e orienta sobre procedimentos de higiene? Realiza testagens?; e condutas: realiza orientações sobre as consultas de enfermagem para acompanhamento das lesões, sobre o tratamento e autocuidado? Oferece testes rápidos? Indica o teste VDRL para a usuária e os comunicantes? Elogia e incentiva o progresso do autocuidado? Estabelece metas de autocuidado? Orienta sobre o tratamento e prevenção? Prescreve o tratamento com penicilina? Preenche a ficha de notificação de sífilis?

4. *Debriefing:* a avaliação - o plano de aula foi testado com um grupo de *experts* composto por docentes, especialistas, enfermeiras(os) e estudantes, sendo que foi avaliado previamente pela banca de juízes e sequencialmente aplicado para ser avaliado pelos estudantes. Após a discussão, em conjunto com os estudantes voluntários e os observadores, os participantes avaliaram a simulação e o plano de aula por meio do questionário semiestruturado.
5. *Assessment:* o plano de aula de simulação foi realizado com estudantes do 7º semestre, acompanhado da aplicação de questionário semiestruturado (escala Likert) para avaliar conteúdo, autoconfiança e percepção no processo de investigação. A atividade, incluindo apresentação, simulação e preenchimento dos questionários, teve duração de aproximadamente 120 minutos.

Foram utilizados como instrumentos de coleta de dados questionários direcionados ao comitê de especialistas, divididos em duas partes: 1) dados sociodemográficos; e 2) itens sobre aspectos técnicos da simulação e conteúdo. Com duração média de 30 minutos, os questionários foram preenchidos durante o teste piloto, antes da aplicação aos estudantes. Inspirados em estudo sobre validação de material didático para a prevenção e manejo da sífilis^(5)^, os questionários avaliaram o método de ensino utilizando a escala Likert, com opções de resposta variando de “discordo plenamente” a “concordo plenamente”.

Foi aplicado questionário sociodemográfico de elaboração própria para os estudantes de graduação. O método de ensino foi avaliado por meio de questionários que utilizam como base a escala Likert, que foi entregue ao término da simulação^(10)^. O questionário dos estudantes abordou a estrutura da simulação, o conteúdo e a autoconfiança do participante.

## RESULTADOS

O comitê de especialistas avaliou positivamente todos os tópicos da aula simulada de investigação de sífilis na gestação. Todos concordaram que a simulação aborda o conteúdo pertinente à temática e que o formato dinâmico pode contribuir significativamente para o aprendizado clínico e epidemiológico. O conteúdo prévio foi considerado pertinente, e o cenário simulado foi avaliado como agradável, informativo, coerente e adequado. Também referiram que o pré-*briefing* e o *debriefing* estão bem detalhados, sendo etapas cruciais para a compreensão e aprendizado da temática. Tal detalhamento, observado em todo o material validado pelo comitê de especialistas, auxilia na quebra de tabus e não fomentam constrangimentos relacionados à temática na consulta de enfermagem.

Quando se avaliaram a satisfação e autoconfiança dos estudantes após a simulação realística, foram elaboradas dez perguntas, e o resultado foi: 63,6% (21) dos participantes concordam plenamente que se sentiram seguros para lidar com a investigação de sífilis na gestação; 87,9% (29) concordam plenamente que a atividade contribuiu significativamente para a própria formação e a apreensão de conteúdos sobre investigação de sífilis na gestação; 78,7% (26) acharam a simulação agradável; 69,7% (23) discordam que preferem atividades de aprendizado tradicionais; 90,9% (30) concordam que eles são capazes de desenvolver habilidades por meio das atividades da simulação; 90,9% (30) concordam plenamente que a explicação inicial, antes do cenário, foi importante para a compreensão da dinâmica; 84,8% (28) concordam plenamente que a discussão da simulação foi eficiente no processo de aprendizado; 93,9% (31) concordam que a simulação ajudou a quebrar tabus em relação à temática da consulta de enfermagem abordada; e 100% (33) concordam que o seu conhecimento sobre a investigação de sífilis na gestação melhorou após a simulação. A única questão que obteve respostas diversas foi sobre o amplo conhecimento sobre a investigação de sífilis antes da simulação, em que obtivemos 60,6% (20) que discordam, 12,1% (4) que não sabem opinar e 27,3% (9) que concordam.

O perfil da amostra apresenta uma composição de maioria branca, com identificação de mulher cisgênero, dividida igualitariamente entre heterossexuais e bissexuais, assumindo ocupação exclusiva como estudante, segundo o levantamento sociodemográfico preenchido pelos participantes.

## DISCUSSÃO

O plano de aula de simulação realística foi validado por um comitê de especialistas e testado por estudantes, destacando a importância de atividades práticas com metodologias de aprendizagem ativa no ensino de enfermagem. A simulação abordou a investigação da sífilis na gestação, essencial para o desenvolvimento do raciocínio clínico dos estudantes, contribuindo para a detecção precoce e tratamento adequado, prevenindo complicações como a SC.

Além de aspectos técnicos, a simulação proporcionou reflexões sobre questões sociais e de saúde pública, como desigualdades no acesso ao cuidado e à informação. O estudo aponta que, ao focar apenas no tratamento técnico, a enfermagem pode negligenciar as implicações sociais da doença, sendo a investigação clínica eficaz, com boa anamnese e avaliação dos riscos, fundamental para prevenir problemas para gestantes e bebês.

Durante o *debriefing*, os estudantes discutiram a autonomia da enfermagem, principalmente em relação à solicitação de exames e prescrição de medicamentos. As discussões indicaram que a consolidação das competências de autonomia e decisão clínica ainda está em desenvolvimento. A simulação ajudou os participantes a refletir sobre suas práticas e limitações na Atenção Primária à Saúde, sugerindo a necessidade de mais estudos sobre a satisfação e autoconfiança dos alunos nesses cenários.

Um aspecto inovador da simulação foi a participação dos pesquisadores, que também eram estudantes, atuando como usuários simulados. Isso proporcionou um ambiente mais confortável e colaborativo, incentivando os estudantes a compartilhar suas opiniões e discutir suas decisões. O *debriefing* foi crucial para a reflexão crítica e consolidação do aprendizado, com a contribuição dos professores como facilitadores do cenário, esclarecendo dúvidas e enriquecendo o processo de ensino.

### Limitações do estudo

Como limitações do estudo, tem-se que ele foi realizado em uma única Instituição de Ensino Superior pública, com amostra pequena e com destaque secundário para o preenchimento da ficha de notificação compulsória. Nesse sentido, os resultados encontrados não podem ser generalizados para estudantes de enfermagem com características distintas da nossa população. O instrumento de captação da satisfação dos estudantes e autoconfiança na aprendizagem foi utilizado somente após a participação na simulação realística.

### Contribuições para a área da enfermagem

Este estudo contribuiu para desenvolver o conhecimento dos estudantes de enfermagem sobre a investigação da sífilis na gestação, aprimorar a qualidade da investigação epidemiológica e capacitar os futuros enfermeiros para realizar consultas de enfermagem de alta qualidade no rastreamento e manejo da sífilis gestacional, utilizando simulação realística. A longo prazo, isso pode contribuir para o aumento da detecção precoce de sífilis na gestação e melhora na prevenção e tratamento da doença, reduzindo os casos de SC e respondendo, assim, aos objetivos propostos pela OMS.

## CONCLUSÕES

O plano de aula desenvolvido e proposto foi avaliado, permitindo que os estudantes obtivessem melhor compreensão da temática de forma aplicada à prática. Nesse sentido, sua contribuição foi evidenciada e poderá ser usada como material para a elaboração didática voltada para doenças transmissíveis e investigação de sífilis na gestação. A proposta de aplicação de metodologia ativa no ensino em enfermagem é promissora e deve ser incentivada, tendo como horizonte a eliminação da SC, conforme os Objetivos de Desenvolvimento Sustentável. A simulação realística aplicada para a investigação de sífilis na gestação configura-se como método de grande aproveitamento didático e acadêmico.

## Data Availability

Os dados de pesquisa só estão disponíveis mediante solicitação.
